# Effect of Buffered Local Anesthesia on Perioperative Pain During Arteriovenous Fistula Surgery: A Randomized Control Trial

**DOI:** 10.7759/cureus.15202

**Published:** 2021-05-23

**Authors:** Anum Arif, Bismah Riaz, Ahsan Manzoor Bhatti, Nawabzada Zeerak Farhat Sherwani, Raoon Khan, Aima Sohail, Aitizaz Shahid, Syed Hashim Ali Inam

**Affiliations:** 1 Vascular Surgery, Combined Military Hospital (CMH) Lahore Medical College and Institute of Dentistry, Lahore, Pakistan, Lahore, PAK; 2 Internal Medicine, Combined Military Hospital (CMH) Lahore Medical and Dental College, Lahore, PAK; 3 Vascular Surgery, Combined Military Hospital (CMH) Lahore Medical College and Institute of Dentistry, Lahore, PAK; 4 Internal Medicine, Combined Military Hospital (CMH) Lahore Medical College and Institute of Dentistry, Lahore, PAK; 5 Medicine, Combined Military Hospital (CMH) Lahore Medical College and Institute of Dentistry, Lahore, PAK; 6 Epidemiology and Public Health, Army Medical College, Rawalpindi, PAK

**Keywords:** pain, local anesthetics, alkalinization, sodium bicarbonate, buffered

## Abstract

Objective: This prospective, pilot randomized double-blind study aimed to compare the effects of buffered and non-buffered xylocaine solutions on injection pain and anesthesia effectiveness in patients undergoing arteriovenous fistula surgery.

Material and methods: A total of 100 adult patients meeting inclusion criteria undergoing arteriovenous fistula surgery were included in the study. They were split into two groups at random. The control group received 1% xylocaine dissolved in 5 ml distilled water, while the intervention group received sodium bicarbonate mixed with 1% xylocaine solution as a local anesthetic. The patients were asked to rate the pain of first and subsequent injections on a visual analog scale (VAS). Besides, the need for extra analgesia was investigated. The mean and standard deviation of the data was determined.

Results: During both the first and subsequent injections, the alkalinized local anesthetic group showed substantially lower VAS scores. In the alkalinized local anesthetic group, anesthesia satisfaction was also more than three times higher. Furthermore, the non-alkalinized group's mean analgesic requirement was higher than the intervention group.

Conclusion: Our findings support the effectiveness of the alkalinized local anesthetic solution in minimizing injection pain and increasing anesthesia duration and overall patient's surgical experience in terms of anesthesia satisfaction score.

## Introduction

A patient with end-stage renal failure requires hemodialysis access most of all [1]. Arteriovenous fistulas (AVF) have the lowest rates of morbidity and mortality of all the vascular access alternatives [2]. The Kidney Disease Outcomes Quality Initiative (KDOQI) guidelines have emphasized the arteriovenous fistula as the first initiative because of its superior patency, lower complication rate, and lower healthcare expense [3]. However, there are several obstacles to this practice including delayed referrals to doctors, patient noncompliance with surgical procedures, and patient comorbidities [4].

Most vascular surgeons tend to operate under local anesthesia due to the complications and risks involved with other types of anesthesia in such patients. According to Filed et al., 73.9 % of respondents thought AVF was better for their health, but they were hesitant to undergo surgery for a variety of reasons, the most common of which was pain during surgery [4]. Pain from lignocaine injections is influenced by surgical experience, needle gauge, rate and volume of infiltration, temperature, and most importantly, the acidic pH of the solution [5].

Several studies have been conducted on the addition of various agents to local anesthetics to improve their potency and reduce discomfort [6,7]. Alkalising the anesthetic solution with sodium bicarbonate is one of the most effective ways [5,8]. Sodium bicarbonate raises the proportion of uncharged base form of local anesthesia by increasing the pH of the solution closer to physiological pH. This enhances soft tissue dispersion, which improves effectiveness and decreases discomfort associated with local anesthesia. Moreover, when the extracellular pH is increased by the addition of sodium bicarbonate, the intracellular pH decreases due to the diffusion of carbon dioxide which also plays a role in increasing the effectiveness of local anesthesia [9].

The potency, duration of action, and the onset of anesthesia of a local anesthetic are influenced by its structure, concentration, pKa (acid dissociation constant), and tissue pH [10]. The aim of this prospective, randomized, double-blind research was to see if adding sodium bicarbonate to a local anesthetic solution affected injection pain, duration, and efficacy.

## Materials and methods

One hundred patients participated in this prospective, randomized, double-blind study. The study was conducted in the Department of Vascular Surgery over six months from June 1, 2020, to December 30, 2020. The clinical trial number was NCT04610307. All patients scheduled for arteriovenous fistula surgery who follow the inclusion requirements of being 18 to 80 years old, male or female, ASA (American Society of Anesthesiologist) I to III, and undergoing radiocephalic or brachiocephalic fistula under local anesthesia were recruited. Patients under the age of 18 years, patients undergoing redo procedures, pregnant mothers, people allergic to local anesthesia, people with an active site infection at the injection site, and people who could not give informed consent were excluded from the study.

Using a basic random sampling procedure, patients were split into two groups of 50. A total of 100 slips of paper with numbers 1-100 were randomly divided into two groups: control and intervention. Each patient chose a slip and received the medication that had been assigned to them at random. In our study, we made sure that the buffered solution was prepared just before the procedure. After explaining the procedure to the patient, the control group was given 1% xylocaine 5 ml diluted in 5 ml distilled water by injection, and the intervention group received 1% xylocaine diluted in 4 ml distilled water and 1 ml sodium bicarbonate. All procedures were conducted by vascular surgeons. Both the surgeons and patients were unaware of which anesthetic the patient had been given.

The surgeon used a visual analog scale (VAS) score to record the pain intensity at the time of the first injection and subsequent injections on a scale of 1 to 10. Furthermore, the pain severity was determined by the number of times additional analgesia was needed as well as the anesthesia satisfaction score. VAS score was characterized as 0 = no pain, 1-3 = mild pain (pain reported only in response to questioning and without any behavioral signs), 4-6 = moderate pain (pain reported in response to questioning and followed by signs, or pain reported spontaneously without questioning), and 7-10 = severe pain (strong vocal response or response accompanied by grimaces, withdrawal of the arm, or tears). Anesthesia satisfaction score was measured on a format of 4 points Likert scale, including “no satisfaction," “least satisfaction," "incomplete satisfaction," and "complete satisfaction" with a score ranging from 1 to 4, respectively.

The data were obtained as mean values. Statistical Package for Social Sciences (SPSS version 22, IBM Corp., Armonk, NY) was used to analyze the data. A p-value of less than 0.05 was considered statistically significant.

## Results

A total of 100 participants were recruited for the study. The mean age was 48.88 ± 14.14 years. There was male predominance (n= 69) and the majority (n=65) had no previous history of arteriovenous fistula. The brachiocephalic fistula was most common (n= 64). Fifty-one patients had moderate pain at first injection and 54 patients had no pain at subsequent injection.

**Figure 1:**
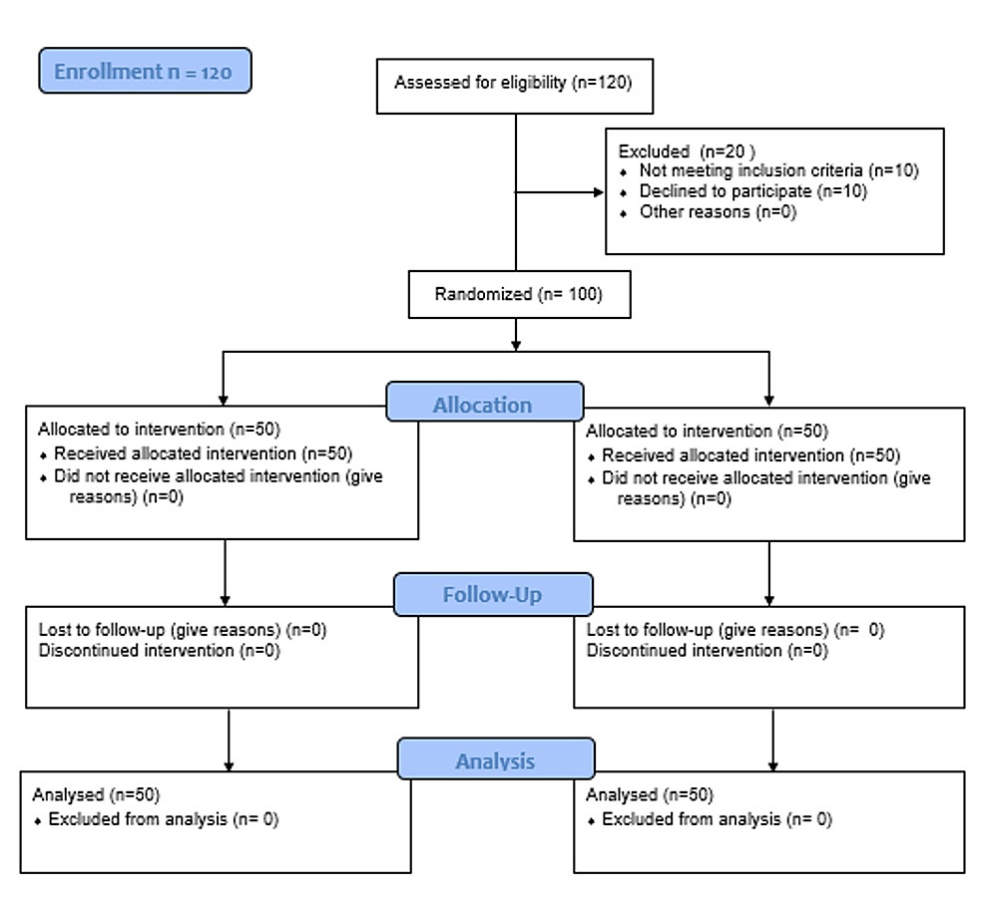
CONSORT diagram of the study

Patients were categorized into control and intervention arms shown as CONSORT diagram in Figure 1.

There was male predominance in both groups (n= 32 and n=37), respectively. Three-quarter of the intervention group and 60% of the control group had no previous history of AVF. The mean pain score at first Injection was 3.8 ± 1.9 and the mean pain score at subsequent injections was 1.5 ± 1.8 (p-value<0.001).

The surgical characteristics of both groups are shown in Table 1.

**Table 1:** Characteristics of surgical procedure, pain, and analgesics in both groups (n=100) AVF: arteriovenous fistulas.

Characteristics	Control group no. (%)	Intervention group no. (%)	p-Value
Type of arteriovenous fistula			
Snuffbox (wrist) AVF	3 (6)	6 (12)	
Proximal radio cephalic AVF	19 (38)	18 (36)	0.577
Brachiocephalic AVF	28 (56)	26 (52)	
Duration of surgery (minutes)			
<30	1 (2)	2 (4)	
31–60	13 (26)	17 (34)	0.567
61–90	27 (54)	26 (52)	
>90	9 (18)	5 (10)	
Additional analgesia required			
Yes	23 (46)	15 (30)	0.099
No	27 (54)	35 (70)	
Frequency of additional analgesia			
No	27 (54)	34 (68)	
Yes, single	12 (24)	7 (14)	0.496
Yes, twice	8 (16)	7 (14)	
>2	3 (6)	2 (4)	

**Figure 2:**
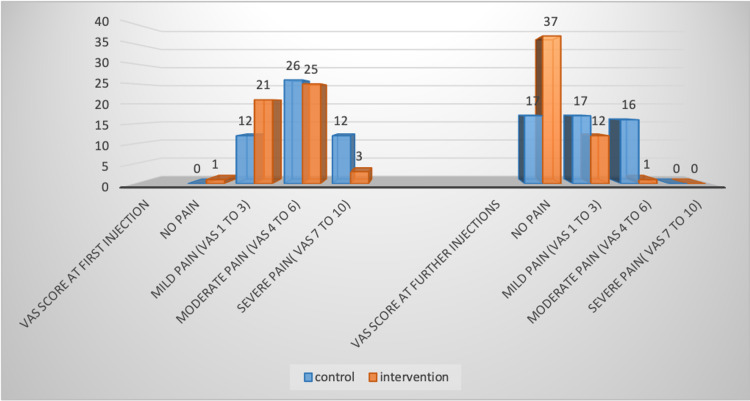
Difference in VAS pain score between either group (n= 100) VAS: visual analog scale.

Figure 2 shows the difference in VAS pain score between either group at the time of first injection and subsequent injection. P-value was 0.03 and <0.001, respectively.

**Figure 3:**
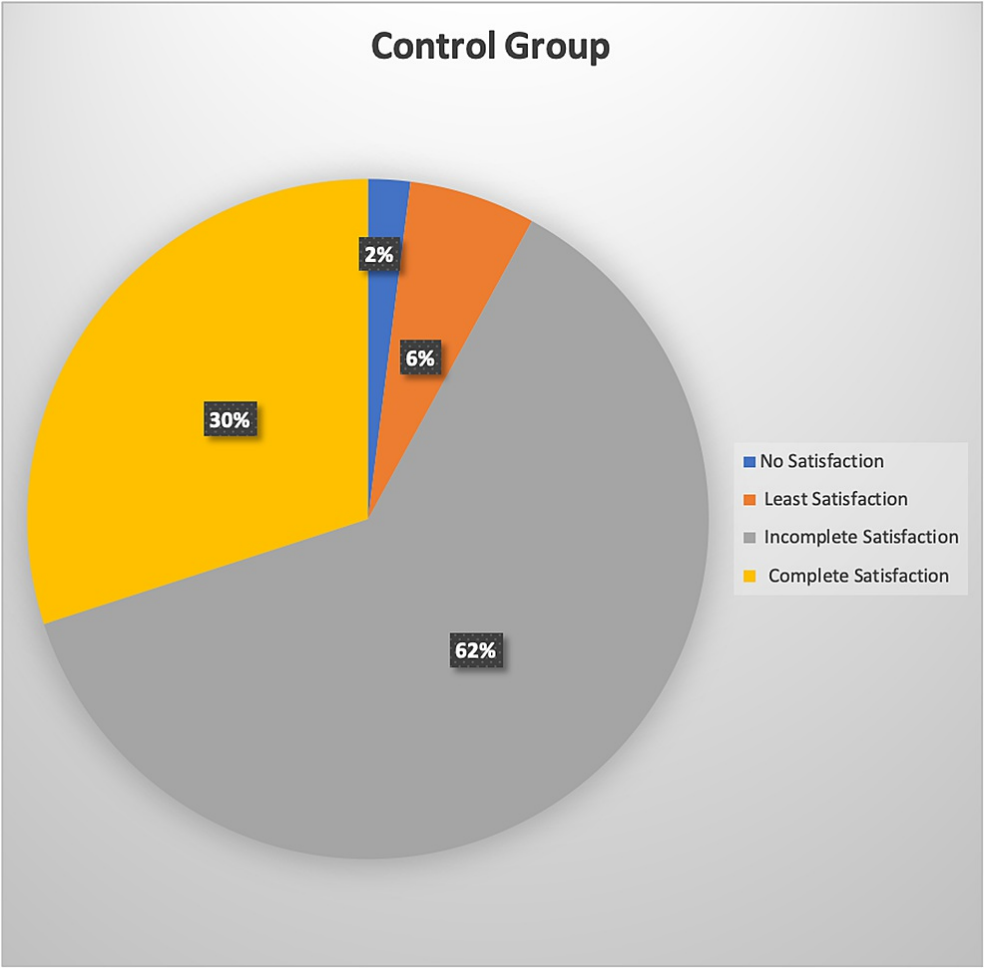
Satisfaction of patients related to anesthesia in the control group

**Figure 4:**
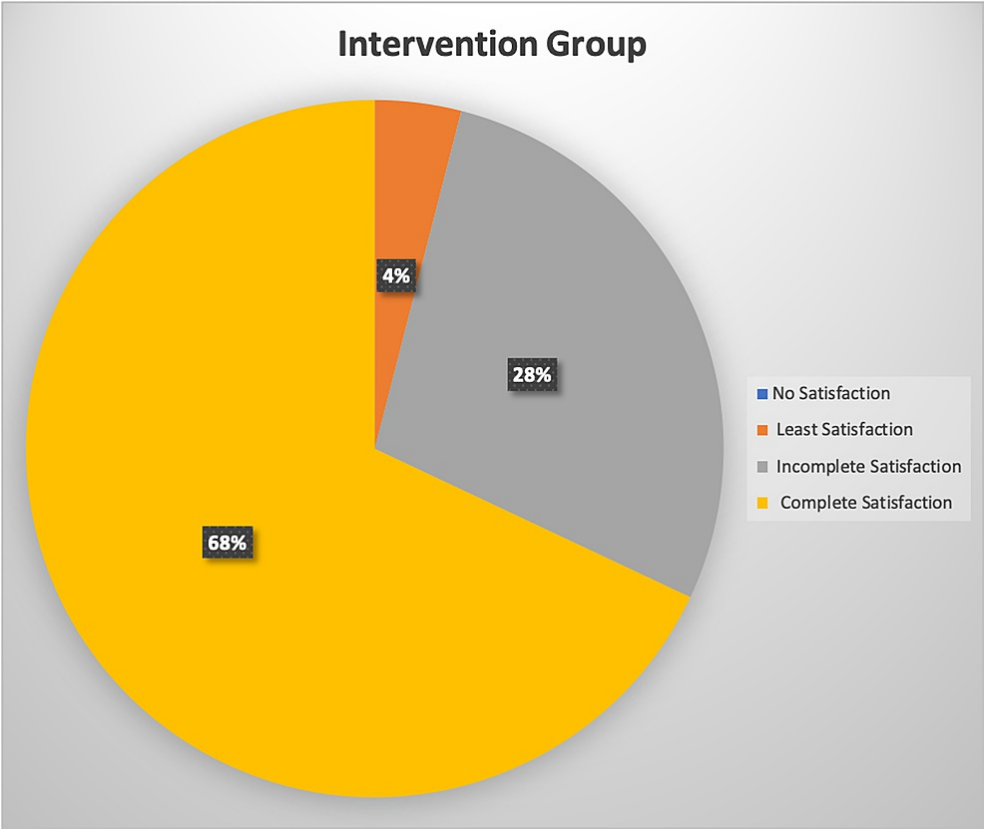
Satisfaction of patients related to anesthesia in the intervention group

Figures 3 and 4 show the anesthesia satisfaction score by the patients at the end of surgery where 1 is no satisfaction at all and it is interpreted as a completely unpleasant experience with the surgical anesthesia and procedure and score 4 is complete satisfaction which is interpreted as an overall pleasant experience with the anesthesia methods used and the surgical experience.

## Discussion

Patients undertaking mild procedures under local anesthesia often feel pain before any surgical intervention due to local anesthesia infiltration [11]. Local anesthetics, when given in appropriate doses, induce transient visual, motor, and autonomic activity loss, as well as electrophysiological changes in nerve fibers, neutrons, and muscle cells. The injection of local anesthetic is uncomfortable for some patients which makes them reject additional procedures requiring local anesthesia, even though it is only temporary. However, it is time-consuming and often contraindicated to give additional analgesics [12]. Therefore, for initial local anesthesia injection to be effective, it should be given slowly and mixed with an alkaline solution [13,14].

There are numerous techniques for minimizing the pain caused by local anesthesia injection, with supporting evidence. One of them is by the addition of sodium bicarbonate to a formulation of lidocaine [5]. Sodium bicarbonate is a systemic alkalizing agent. It is commonly used in the treatment of metabolic acidosis. The acidity of local anesthesia is the main cause behind the pain caused by local anesthesia. When sodium bicarbonate is added to lidocaine, the pH increases to 7.38 which is close to the physiological pH. The basic non-ionized local anesthesia has a higher ability to diffuse across the membrane of the nerve. It results in the early onset of anesthesia and reduced pain during the injection [10,15]. Moreover, carbon dioxide is also produced by sodium bicarbonate [16]. Carbon dioxide increases intracellular acidification and also induces further anesthetic diffusion through the membrane due to the increased concentration gradient of un-ionized anesthetic [17].

There have been various studies that show that buffered local anesthesia is effective in reducing the pain during local anesthesia infiltration [14,18]. Our study is the first study to research the effect of buffered local anesthesia in arteriovenous fistula surgery. In our research, the intervention group receiving buffered local anesthesia felt reduced pain at first injection (p-value=0.024) and almost no pain at subsequent injections (p-value<0.001). Therefore, our results are consistent with the findings of previous studies on buffered local anesthesia but in other surgical procedures. Some researchers contradict this theory as they found no significant change in the intensity of pain using buffered anesthesia [19,20].

Some reports claim that buffering of local anesthesia prolongs the duration of local anesthesia [21,22]. In our study, we analyzed the duration of anesthesia between the two groups by checking for additional analgesia requirements. The group using buffered anesthesia required relatively fewer analgesics compared to the control group. However, the difference was not statistically significant (p-value=0.099). The duration of the effect of local anesthesia depends on the affinity and rate at which local anesthetics bind to protein receptors in the sodium channels of nerve membranes. Additionally, the vascularity of the administration site may also affect the duration of anesthesia [10]. Some researchers like Christoph et al. [23] concluded that buffering did not affect the duration of anesthesia, unlike our study.

Although alkalinization of local anesthetic provides early onset and a reduction in pain, it may cause precipitation [24]. This could reduce its bioavailability and impair its effectiveness. Therefore, buffering of local anesthetics should be done immediately before administration [25]. In our study, we made sure that the buffered solution was prepared just before the procedure. This helped preserve the potency of the anesthetics.

Satisfaction is the balance between prior expectation and later perception of a health care received. The poor quality will resist patients from using the service with comfort as it should be. In our study, we found that none of the participants in the intervention group and 2% in the control group reported no satisfaction with the surgical anesthesia whereas 68% in the intervention group and 30% in the control group had complete satisfaction with surgical anesthesia. This further emphasizes the non-inferiority of buffered lignocaine as a standard local anesthetic for AVF surgery (p-value=0.02). Hence, patients would be more likely to follow fistula first practice to some degree as a result of increased satisfaction.

There were, however, certain limitations to our study. It is a single-center study with a limited number of patients. Multicenter trials are required to validate our results further. Moreover, different people have different tolerance to pain. This limited our ability to fully study the effect of buffered solutions on the duration of anesthesia.

In our study, the VAS score at first and at subsequent injections was significantly reduced in the intervention group (p-value=0.031 and p-value<0.001, respectively, for the first injection and subsequent injections). This reflects the hypothesis that local anesthetics are more potent in alkaline solutions than in neutral or acidic solutions for nerves with intact sheaths [13,26]. However, some articles [19,20] dispute this theory, so further analysis on the subject is required.

## Conclusions

End-stage renal disease patients are often elderly and weak, with various co-morbidities, and unfit for general anesthesia. Angio access surgery is best conducted under local anesthesia, and all attempts should be taken to make it a pleasant experience for them. The pain of infiltration of local anesthesia can be significantly reduced by buffering their pH close to physiological pH. Buffering of local anesthesia solutions is associated with reduced pain during injection, early onset of anesthesia, and prolonged duration of analgesia. It has little to no impact on surgical results, but it does increase patient comfort during the surgery. Buffered local anesthesia is a quick and cost-effective alternative that is often ignored during procedures. Therefore, further research is needed to shed light on this subject.
